# NKCC1: A key regulator of glioblastoma progression

**DOI:** 10.1002/1878-0261.70242

**Published:** 2026-03-24

**Authors:** Anja Thomsen, Diana Freitag, Madlen Haase, Christian Senft, Falko Schwarz, Silke Keiner

**Affiliations:** ^1^ Department of Neurology Jena University Hospital‐Friedrich‐Schiller University Jena Jena Germany; ^2^ Department of Neurosurgery Jena University Hospital‐Friedrich‐Schiller University Jena Jena Germany; ^3^ Experimental Trauma Surgery, Department of Trauma, Hand and Reconstructive Surgery Jena University Hospital, Friedrich Schiller University Jena Jena Germany; ^4^ Jena Centre for Healthy Aging Jena University Hospital Jena Germany

**Keywords:** chloride homeostasis, GABAergic signaling, KCC2, tumor recurrence

## Abstract

Glioblastoma (GBM) is the most common and aggressive primary brain tumor in adults, with poor prognosis despite multimodal therapy. Chloride cotransporters NKCC1 and KCC2 are key regulators of intracellular chloride levels and thereby determine whether GABA acts inhibitory or excitatory. In GBM, disrupted chloride homeostasis promotes proliferation, migration, and stem‐like properties, but its clinical relevance is not fully understood. We analyzed *NKCC1* and *KCC2* expression in GBM samples, considering clinical parameters, such as age, gender, and MGMT promoter methylation. Statistical analyses included ROC‐based cutoff determination, Kaplan–Meier survival analysis, and subgroup. Immunohistochemistry was performed to identify cell types expressing NKCC1. *NKCC1* expression was significantly higher in older patients and emerged as a prognostic marker for recurrence‐free survival, with lower levels correlating with delayed recurrence, although overall survival was unaffected. *NKCC1* was expressed in stem‐like, astrocytic, and neuronal progenitor cells, but not in mature neurons. These findings identify *NKCC1* as a regulator of GBM progression and recurrence, linking chloride transporter imbalance to GABAergic signaling. Targeting *NKCC1* and restoring chloride homeostasis may provide promising new treatment strategies.

## Graphical abstract

Glioblastoma (GBM) progression is driven by disrupted chloride cotransporter homeostasis. NKCC1 is highly expressed in stem‐like, astrocytic, and progenitor cells, correlating with earlier recurrence, while overall survival remains unaffected. NKCC1 serves as a prognostic marker and potential therapeutic target, linking chloride transporter imbalance to GABAergic signaling. Restoring chloride homeostasis may offer novel GBM treatment strategies.

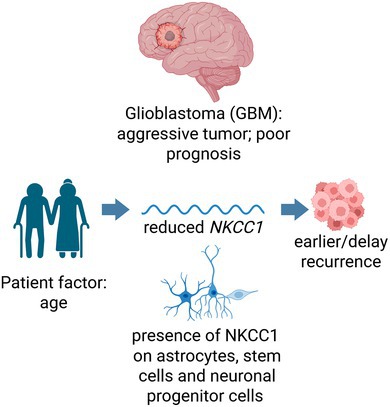

**Abbreviations**
cDNA: Complementary DNA
Ct Cycle: Threshold (qPCR measurement)
DCX: Doublecortin (neuronal progenitor marker)
FFPE: Formalin‐fixed, paraffin‐embedded
GBM: Glioblastoma
IDH: Isocitrate dehydrogenase
KCC2: Potassium‐Chloride Cotransporter 2
MGMT: O6‐Methylguanine‐DNA Methyltransferase
NDS: Normal donkey serum
NeuN: Neuronal nuclei (marker for mature neurons)
NKCC1: Sodium‐Potassium‐Chloride Cotransporter 1
OS: Overall survival
PBS: Phosphate‐buffered saline
PFS: Progression‐free survival
qPCR: Quantitative polymerase Chain reaction
ROC: Receiver operating characteristic
S100b: S100 Calcium Binding Protein B (astrocyte marker)
TERTp: Telomerase reverse transcriptase promoter

## 1 Introduction

Glioblastoma (GBM) is the most common and most aggressive primary brain tumor in adults [1]. Despite intensive multimodal treatment approaches, including surgical resection, radiotherapy, and chemotherapy with temozolomide, the prognosis for patients remains extremely poor. The median survival time is approximately 15 months after diagnosis [2] and the five‐year survival rate is less than 5% [3]. This unfavorable outcome is largely driven by the high invasiveness of the tumor, its genetic and epigenetic heterogeneity, and the ability of tumor cells to evade therapeutic strategies and develop resistance. Consequently, a central challenge in GBM research is to identify molecular mechanisms that regulate tumor growth, invasiveness, and therapy resistance in order to develop novel therapeutic strategies. In this context, ion channels and transporters have received increasing attention, as they are indispensable for maintaining cellular homeostasis, regulating cell volume, and mediating signal transduction.

Among these, the cation‐chloride cotransporters NKCC1 and KCC2 are of particular interest. They are essential regulators of intracellular chloride concentration and play a pivotal role in the functional development of the central nervous system. NKCC1 is mainly active in immature neurons, where it increases intracellular chloride levels, whereas KCC2 is expressed in mature neurons and lowers chloride concentration by extruding ions [1, 4, 5]. Through their opposing functions, these transporters shape the action of the neurotransmitter GABA, which depolarizes immature neurons but hyperpolarizes mature ones [6, 7, 8, 9].

In GBMs, dysregulation of chloride transport appears to contribute directly to tumor progression. NKCC1, in particular, has emerged as a critical factor in glioma biology [5, 10, 11, 12, 13, 14]. By mediating chloride influx, NKCC1 facilitates changes in cell volume and cytoskeletal organization that are essential for GBM cells to migrate through the dense extracellular matrix of the brain. Upregulated NKCC1 activity has been associated with enhanced proliferation, invasiveness, and survival of GBM cells [4]. Pharmacological inhibition of NKCC1 has been shown in experimental models to reduce glioma cell migration and invasiveness, highlighting its potential clinical relevance [15, 16, 17]. Given its dual role in regulating both chloride homeostasis and GBM cell behavior, NKCC1 represents a promising candidate for therapeutic intervention aimed at limiting tumor progression and improving patient outcomes.

The role of NKCC1 in GBM and its potential therapeutic implications highlight the relevance of these transporters for neuronal function and the pathogenesis of neurological and oncological diseases.

## 2 Materials and methods

### 2.1 Human samples

Transcriptional analyses were performed on 61 GBM samples surgically obtained from the Department of Neurosurgery, University Hospital Jena between October 2010 and April 2022. Of the 61 samples, the measurement results for *NKCC1* and *KCC2* were below the measurement limit in six samples, which is why the following results refer to 55 samples. In addition, 10 FFPE samples were used for the localization and colocalization of specific cell types by immunofluorescence staining and confocal microscopy; these samples were provided by the Institute of Neuropathology, Charité—University Medicine Berlin. All patients were informed about the purpose of tissue collection and provided written informed consent for the pseudonymized storage and use of their samples after the procedures had been explained in detail. The study involving human tissue from intracranial tumors taken directly for the study or from the biobank of the Clinic for Neurosurgery in Jena was approved by the local ethics committee for clinical studies at Friedrich Schiller University in Jena (reg. no. 2019–1400 and 2018–1144) and conducted in accordance with the Declaration of Helsinki. The experiments were undertaken with the understanding and written consent of each subject. Glioblastoma multiforme (GBM) is the most common malignant primary brain tumor in adults, accounting for 50.1% of cases [3], and represents a poor prognosis for affected patients. According to the WHO classification, it is classified as a diffuse glioma and, due to its rapid and infiltrative growth, as WHO Grade 4, together with isocitrate dehydrogenase (IDH)‐mutated astrocytomas and IDH‐mutated and 1p/19q codeleted oligodendrogliomas. In diffuse gliomas without IDH mutation and without nuclear ATRX loss, pathological vascular proliferation and/or necrosis and/or at least one of the following changes, such as a mutation in the TERT promoter (TERTp), EGFR amplification, or + 7/−10 copy number alteration are required for classification as GBM [18]. In this study, only tumor samples that could be clearly classified as GBM based on morphological and molecular diagnostic criteria were examined (Table S1).

### 2.2 Total RNA extraction, reverse transcription and quantitative polymerase chain reaction

Surgically removed tissue was immediately stored at −80°C until processing. Total RNA from the tissue sample was isolated, lysed in 1 mL Qiazol (Qiagen GmbH, Hilden, Germany), and homogenized with TissueLyser LT (Qiagen GmbH, Hilden, Germany) at 50 Hz for 5 min. The lysed tissue sample was mixed with 0.2 volume units (PU) of chloroform and centrifuged at 12000 × ***g*** at 4 °C for 15 min. The upper aqueous RNA phase was removed and mixed with 1 PU isopropanol and centrifuged at 12000 × ***g*** at 4 °C for 10 min. The resulting pellet was dissolved twice in 75% ethanol and precipitated at −20 °C for 1 h. The resulting pellet was air‐dried for 10 min and resuspended in 40 μL RNASE‐free water.

The RNA quality and concentration was determined spectrophotometrically (NanoDrop2000; Thermo Scientific, Wilmington, DE, USA). Complementary DNA synthesis was performed using the GoScriptTM Reverse Transcription System (Promega Corp., Mannheim, Germany) in a volume of 20 μL per reaction according to the manufacturer's instructions.

The prepared cDNA was used as the basis for qPCR with the DyNAmo Flash SYBR Green qPCR Kit (Thermo Scientific Inc., Wilmington, DE, USA) to determine the mRNA levels of the transporters *NKCC1* and *KCC2*. The human‐specific primers used for qPCR were designed based on mRNA coding sequences (NCBI Nucleotide) with NetPrimer (PREMIER Biosoft, Palo Alto, CA, USA) and NCBI Primer‐BLAST and checked for hairpins and other secondary structures. The specificity of the amplification reaction was analyzed by gel electrophoresis and melting curve analysis.

The primer sets are listed in Table 1. The specific transcripts were amplified in a RotorGeneG (Qiagen GmbH, Hilden, Germany) with the following program: 7 min polymerase activation, 40 amplification cycles (95 °C for 10s, 55 °C for 20s, and 72 °C for 30s). To quantify the expression of target genes, we normalized the Ct value using *RPL13A* and *CYC1* as reference genes and normalized expression using the efficiency‐based ∆Ct method (Pfaffl, 2001).

**Table 1:** qPCR primers (fw: forward, rv: reverse).

Gene	sequence 5′→3′	Accession number	Product (bp)
*CYC1*	fw: GAG GTG GAG GTT CAA GAC GG rv: TAG CTC GCA CGA TGT AGC TG	NM_001916.5	160
*KCC2*	fw: CGA AAT CCT GCT GGC TTA CC rv: AGA GGA TGA CAC AAC CCA GG	NM_020708.5	197
*NKCC1*	fw: AAG ACC AAG ACA TAC CGG CA rv: GGA TTG GTG GTA GGT CC	NM_001256461.2	169
*RPL13A*	fw: CCT GGA GAA GAG GAA AGA GA rv: TTG AGG ACC TCT GTG TAT TTG TCA A	NM_001270491.2	126

### 2.3 Immunofluorescence staining

The FFPE sections were deparaffinized by a descending ethanol series. Antigen retrieval was performed by incubating citrate buffer (pH 6.0) at 60 °C for 30 min. After washing with phosphate‐buffered saline (PBS), the nonspecific binding sites were blocked with 20% normal donkey serum (NDS) for 120 min. Hybridization of the primary antibodies: rabbit anti‐NKCC1 (Proteintech™, Manchester, UK; 1 : 250), chicken anti‐DCX (Abcam, Cambridge, UK; 1 : 500), mouse anti‐nestin (Abcam; 1 : 500), mouse anti‐NeuN (Merck Millipore KGaA, Darmstadt, Germany; 1 : 500), and/or mouse anti‐S100b (Sigma Aldrich Chemie GmbH, Taufkirchen, Germany; 1 : 500) was performed overnight at 4 °C. The next day, the slides were incubated at 4 °C. After two washes, the secondary antibodies were hybridized: Alexa488 donkey anti‐mouse (Jackson Immunoresearch, West Grove, PA, USA; 1 : 200), Alexa488 donkey anti‐chicken (Jackson Immunoresearch; 1 : 200), Cy5 donkey anti‐mouse (Jackson Immunoresearch; 1 : 200), and Rhodamine donkey anti‐rabbit (Jackson Immunoresearch; 1 : 200) for 2 h. Nuclear staining was performed with DAPI.

### 2.4 Colocalization of NKCC1 with stem cell, glial, progenitor and neuronal marker using immunofluorescence staining

Ten GBM samples were used for fluorescent immunohistochemistry. Tissue sections were pre‐embedded in paraffin and provided by Institute of Neuropathology, Charité‐University Berlin. Colocalization analysis was performed for NKCC1 with markers of stem cells (Nestin), mature astrocytes (S100b), neuronal progenitor cells (Doublecortin, DCX), and mature neurons (NeuN).

Fluorescence imaging was performed using the Keyence microscope at 4 × magnification for overview images. High‐magnification images (40 ×) were acquired using the LSM980 confocal microscope (Zeiss, Jena, Germany). Individual cells were also imaged to confirm the specificity of the staining.

### 2.5 Statistics

Statistical analyses were carried out using SPSS software (version 23). Gene expression (qPCR) data were first tested for normal distribution. Depending on the distribution, differences between two groups were analyzed using the Mann–Whitney *U*‐test.

For survival analysis, receiver operating characteristic (ROC) curves were generated in SPSS, and an optimal cutoff value was determined using the Youden index (J = sensitivity + specificity – 1). This cutoff was applied to stratify normalized expression values for subsequent analyses. Progression‐free survival (PFS) and overall survival (OS) were evaluated using Kaplan–Meier survival analysis. A p‐value of ≤ 0.05 was considered statistically significant.

## 3 Results

### 3.1 Age‐dependent increase of NKCC1 in glioblastoma

**Fig. 1:**
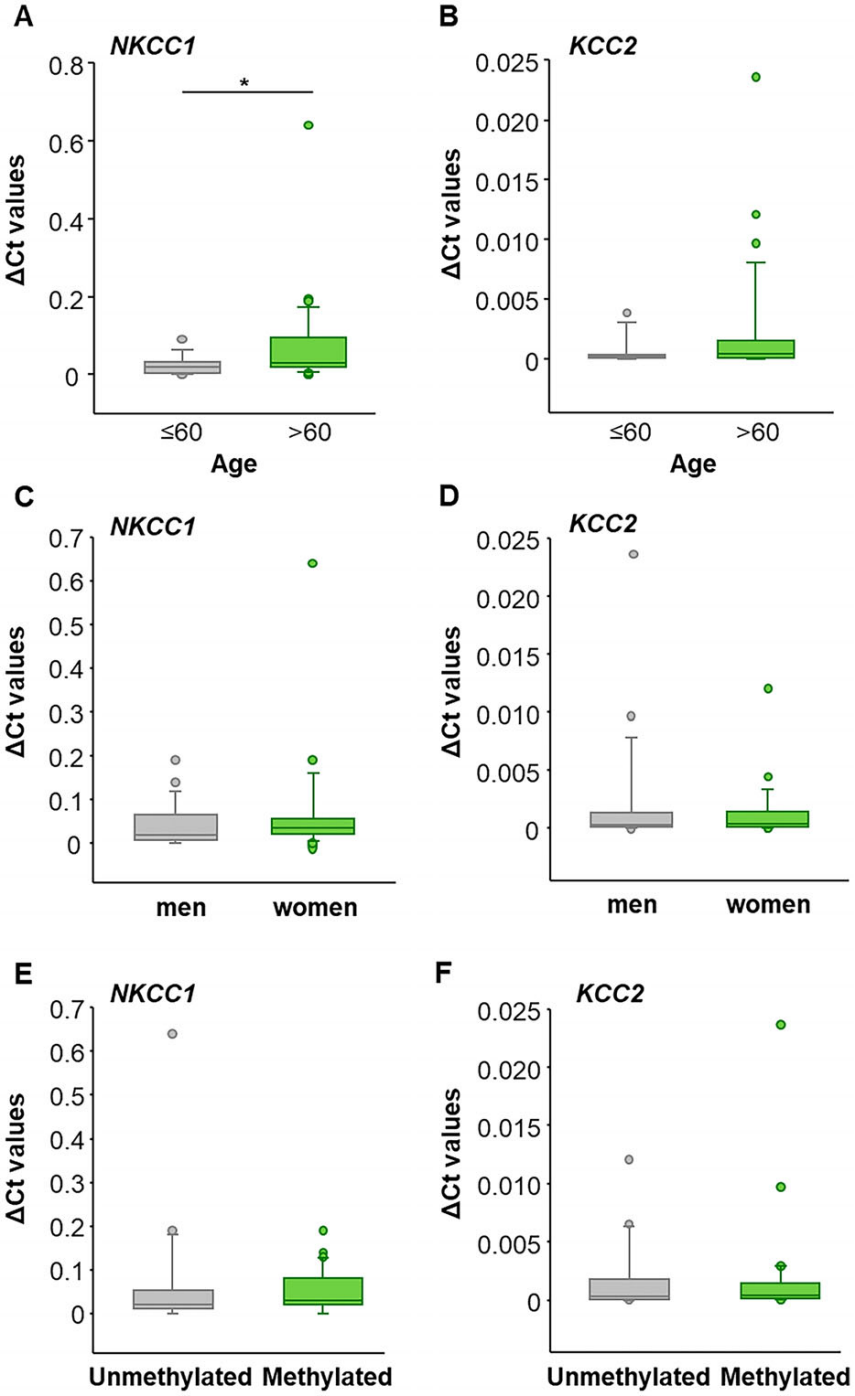
Expression of NKCC1 and KCC2 in relation to clinical parameters: age, gender, and methylation status. (A) *NKCC1* was expressed significantly more strongly in individuals over 60 years of age compared with those under 60 (*n* = 50 biological replicates with technical duplicates). (B) There were no age‐related differences in *KCC2* expression in glioblastoma (GBM) (*n* = 51 biological replicates with technical duplicates). The expression of *NKCC1* and *KCC2* was not affected by C/D sex or E/F methylation status. Box plots show the median and interquartile range (25th–75th percentile) of ΔCt values. Statistical analysis was performed using the Mann–Whitney *U*‐test. *p ≤ 0.05 was considered statistically significant.

The patients' age, sex, and MGMT methylation status were analyzed (Fig. 1). Significant differences were observed in *NKCC1* expression based on age: younger patients (≤ 60 years) exhibited approximately threefold lower NKCC1 expression (mean = 0.02374) compared with older patients (> 60 years) (mean = 0.07220), which was statistically significant (*p* = 0.027) (Fig. 1A,B).

Regarding sex, *NKCC1* and *KCC2* expression levels were similar between males and females (*NKCC1*: males mean = 0.04089, females mean = 0.06978; *KCC2*: males mean = 0.00213, females mean = 0.00129) (Fig. 1C,D). Likewise, no significant differences were found based on MGMT methylation status (*NKCC1*: unmethylated promoter mean = 0.06367, methylated promoter mean = 0.04881; *KCC2*: unmethylated promoter mean = 0.00164, methylated promoter mean = 0.00175) (Fig. 1E,F). In contrast, KCC2 expression showed no age‐dependent differences, with comparable mean expression levels in younger patients (≤ 60 years; mean = 0.00066) and older patients (> 60 years; mean = 0.00223; *p* = 0.155).

### 3.2 Decreased NKCC1 levels correlate with progression‐free survival

**Fig. 2:**
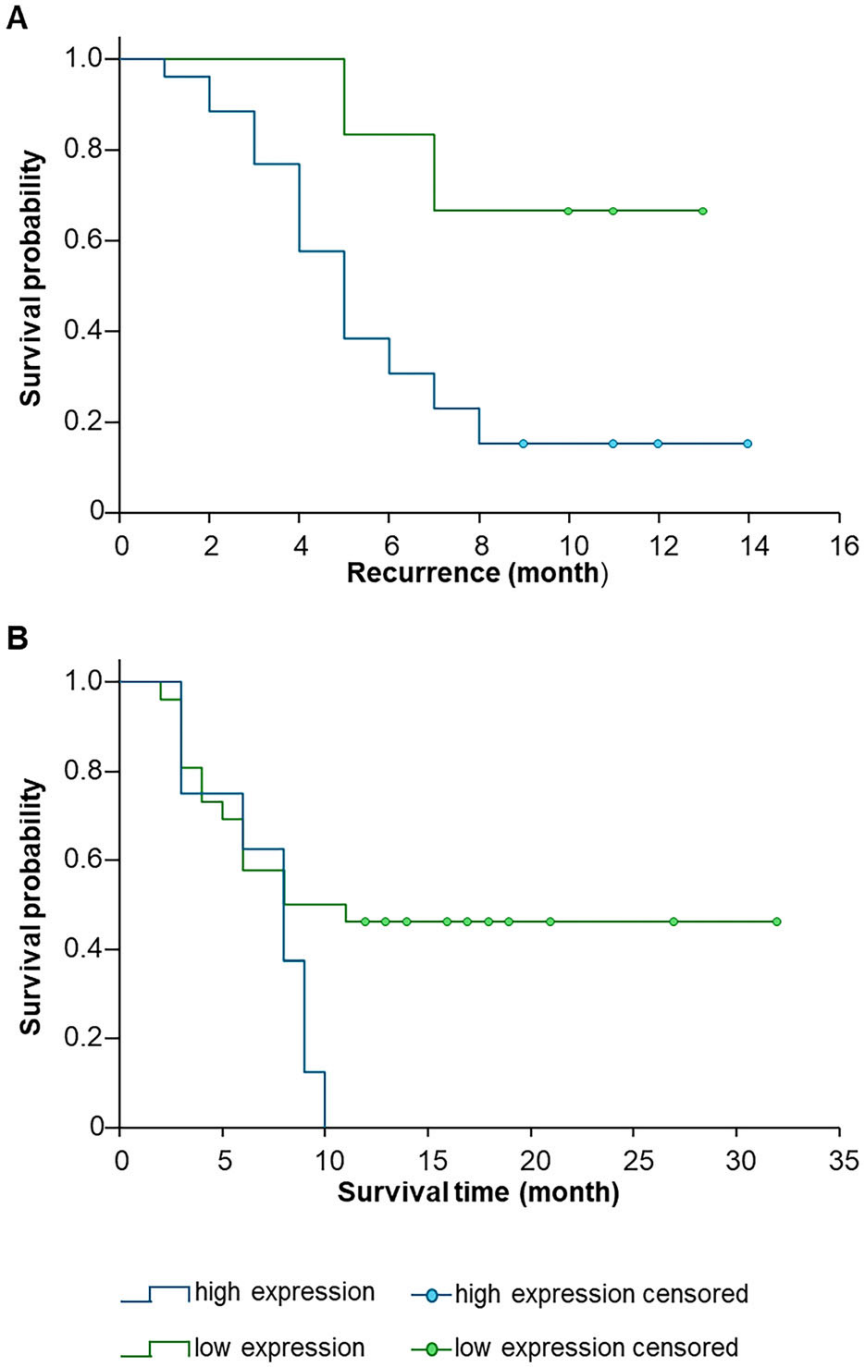
Progression‐free and overall survival (OS) in relation to *NKCC1* expression. (A) Kaplan–Meier analysis showing that patients with low *NKCC1* expression had significantly longer progression‐free survival after 9 months. Forty‐eight patient samples with technical duplicates and mean ΔCT values were used and the difference between groups was evaluated using the log‐rank test (*p* ≤ 0.05). (B) OS tended to be longer in patients with low *NKCC1* expression after 12 months, but this difference was not statistically significant (log‐rank test, *p* > 0.05).

To assess the impact of *NKCC1* expression on recurrence‐free survival, a cutoff value of NE = 0.01401 was determined for individual patients (*n* = 48) using ROC analysis (Fig. 2A). Kaplan–Meier analysis demonstrated that patients with *NKCC1* expression below this cutoff (NE < 0.01401) had significantly longer recurrence‐free survival during the first 9 months after surgery compared to patients with higher *NKCC1* expression (NE > 0.01401; *p* = 0.019) (Fig. 2A).

For OS analysis, 43 patients were included (16 patients excluded due to missing death data and two patients due to long‐term survival > 60 months), and a cutoff value of NE = 0.04028 was established (Fig. 2B). Kaplan–Meier analysis revealed no significant association between *NKCC1* expression and OS (log‐rank test; *p* = 0.078). Kaplan–Meier analysis based on gender revealed no differences in both OS and PFS. In contrast, when stratified by age, the analysis showed differences in OS but not in PFS (Fig. S1).

### 3.3 NKCC1 colocalization with specific glial and progenitor cell types in glioblastoma

Ten GBM samples were subjected to fluorescence staining and subsequently imaged at both 4 × and 40 × magnifications for visual evaluation. NKCC1 was stained alongside various glial and neuronal markers, including Nestin, S100b, DCX, and NeuN. Nestin served as a marker for neural stem cells, S100b for astrocytes, DCX for neuronal progenitor cells, and NeuN for mature neurons.

**Fig. 3:**
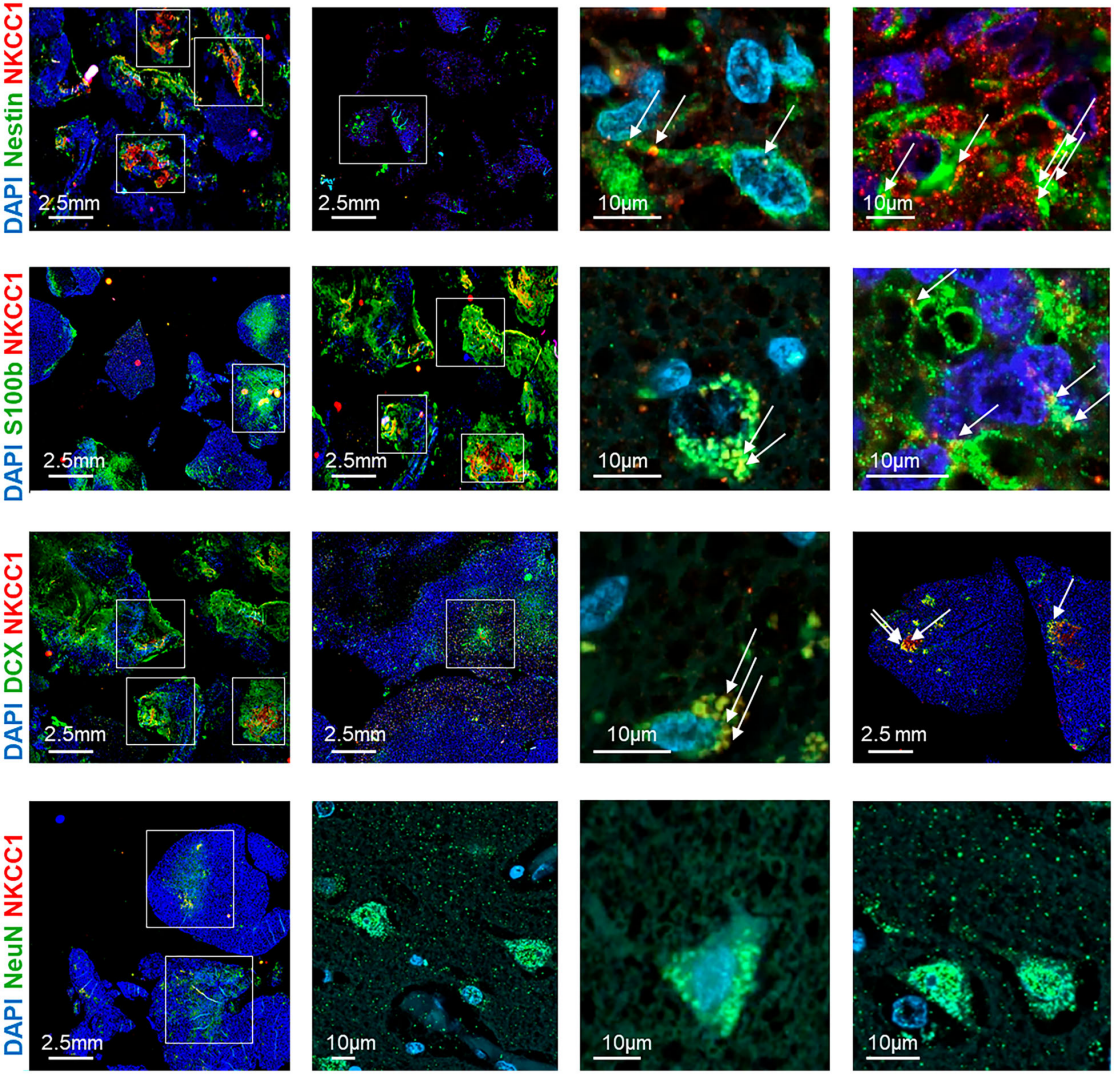
Immunohistochemical analysis of glioblastoma (GBM) samples showing NKCC1 expression in different cell types. The first two columns show stained GBM samples at 4x magnification (scale bar: 2.5 mm). These overview images illustrate the clustering behavior of different cell types: stem cells, astrocytes, neuronal progenitor cells, and mature neurons each co‐stained with NKCC1. Clear colocalization of NKCC1 (red) was observed with Nestin‐positive stem cells, S100b‐positive astrocytes, and DCX‐positive neuronal progenitor cells (green) and DAPI (blue). In contrast, no colocalization was detected with NeuN‐positive mature neurons (green). Images in the third and fourth columns, acquired at 40 × magnification (scale bar: 10 μm), provide higher‐resolution views of NKCC1 expression (red) in various cell types (green) within GBM tissue. White squares indicate NKCC1‐positive cells in the GBM tissue. White arrows mark the presence of NKCC1 in the different cell types.

Overview images at 4 × magnification revealed a heterogeneous distribution of the labeled cell types throughout the tissue (Fig. 3). Rather than a uniform pattern, all samples displayed distinct regional clusters of specific cell populations. The fluorescence intensity of each marker protein (Nestin, S100b, DCX, or NeuN) was notably increased in defined areas, indicating local accumulations of the respective cell types.

High‐resolution imaging at 40x magnification demonstrated colocalization of the chloride cotransporter NKCC1 with multiple cell types within the GBM tissue (Fig. 3). A pronounced overlap was observed between NKCC1 signals and Nestin‐positive cells, suggesting expression in neural stem cells. Additionally, colocalization occurred with S100b‐positive astrocytes and DCX‐positive neuronal progenitor cells. Conversely, no significant colocalization was detected between NKCC1 and NeuN‐positive mature neurons.

## 4 Discussion

The present study examined the expression and potential role of the chloride cation cotransporters NKCC1 and KCC2 in human GBMs, taking into account clinical parameters, such as age, sex, and MGMT promoter methylation status. The results contribute to understanding the molecular heterogeneity of GBMs [19] and suggest possible implications for prognosis and therapy.

An age‐dependent increase in NKCC1 expression was observed, indicating a potential association between patient age and transporter activity. In GBM, *NKCC1* (SLC12A2) has been implicated in key cellular processes, such as ion homeostasis, cell‐volume regulation, and tumor cell migration, all of which are critical for the highly infiltrative growth pattern of this disease. In our cohort, higher *NKCC1* expression was observed in older patients and was associated with an increased risk of tumor recurrence; however, *NKCC1* expression did not translate into a significant impact on OS.

This apparent discrepancy can be explained by the fact that tumor recurrence is nearly universal in GBM and that OS is predominantly determined by established clinical and molecular factors, including extent of resection, MGMT promoter methylation status, and treatment intensity. Consequently, while elevated *NKCC1* expression may contribute to more frequent recurrence, its influence on OS is likely outweighed by these stronger prognostic determinants. Mechanistically, several lines of evidence support a role for NKCC1 as a driver of GBM proliferation and migration through its regulation of intracellular chloride levels and cell‐volume dynamics [5, 11, 12, 17]. By mediating the co‐transport of Na^+^, K^+^, and Cl^−^ into the cell, NKCC1 increases intracellular osmolarity, thereby promoting water influx and controlled cell swelling [20, 21, 22]. These cyclical volume changes are essential during the G2/M transition, when glioma cells must undergo coordinated expansion and condensation to enable chromosome segregation and successful mitosis. Enhanced NKCC1 activity can therefore facilitate these biophysical requirements and promote increased proliferative capacity in GBM cells. The contribution of NKCC1 to tumor cell migration is similarly volume‐dependent. Infiltrating glioma cells must dynamically deform their cytoplasm to traverse narrow extracellular spaces within the brain. NKCC1‐mediated ion influx at the leading edge generates osmotic water entry and localized cell expansion, enabling forward protrusion of the cell body. At the trailing edge, coordinated efflux of K^+^ and Cl^−^, together with aquaporin‐mediated water release, supports cell‐volume reduction and retraction. In addition, NKCC1‐dependent alterations in intracellular Cl^−^ concentrations modulate actin dynamics, cytoskeletal tension, and cell polarity, collectively enhancing motility and invasiveness. Consistent with this, genetic or pharmacological inhibition of NKCC1 has been shown to reduce glioma cell migration and invasion *in vitro* and *in vivo*. Beyond its role in ion transport, NKCC1 also influences cytoskeletal organization. Its silencing leads to decreased F‐actin content, altered actin architecture, reduced activity of the small Rho GTPases RhoA and Rac1, impaired focal adhesion dynamics, and diminished cell contractility, all of which further limit migratory capacity. Taken together, NKCC1 supports both mitosis‐associated volume cycling and the ion‐driven cellular deformation required for motile behavior. Upregulated NKCC1 expression may therefore directly enhance proliferation and invasiveness two hallmarks of GBM that critically contribute to early tumor recurrence. These findings suggest that NKCC1 primarily functions as a marker and mediator of invasive, recurrence‐prone tumor characteristics in GBM rather than as an independent prognostic factor for OS.

This observation may partly explain the generally more aggressive tumor behavior observed in older patients [5, 15].

*NKCC1* also emerged as a potential marker for PFS. Patients with lower *NKCC1* expression exhibited longer progression‐free intervals, while OS was not significantly associated with *NKCC1* levels. These findings suggest that *NKCC1* gene expression may be particularly relevant for early tumor progression and recurrence rather than OS. From a translational perspective, *NKCC1* represents a potential therapeutic target. Current inhibitors, such as bumetanide, are limited by side effects and poor brain penetration, highlighting the need for the development of more selective and brain‐penetrant compounds [15, 17].

GABA is the main inhibitory neurotransmitter in the mature brain, acting through GABA_A_ and GABA_B_ receptors to hyperpolarize neurons and suppress excitability. GBM cells, however, retain functional GABA receptors and are responsive to GABA signaling, which can influence their electrophysiological state and cellular behavior. In glioma cells, GABA can induce membrane depolarization or hyperpolarization depending on the intracellular chloride concentration, which is determined in part by chloride transporters, such as NKCC1 and KCC2 [23].

Excitatory GABA signaling in GBM can modulate intracellular calcium levels, alter ion homeostasis, and affect electrical network properties within the tumor [24]. These effects may influence how glioma cells interact with the surrounding microenvironment and with each other, shaping their electrophysiological behavior independently of classical neuronal inhibition. Studies have shown that GBM cells respond to GABA stimulation in a context‐dependent manner, highlighting that GABA acts as an important modulator of tumor cell physiology rather than merely a neurotransmitter.

Overall, GABA in GBM plays a complex role, capable of modulating membrane potential, calcium signaling, and intercellular communication within the tumor. Its effects depend on the intracellular chloride environment, but the key point is that GBM cells remain sensitive to neurotransmitter signaling, which can influence tumor physiology and network dynamics.

Immunohistochemical analyses demonstrated cell type‐specific NKCC1 expression in GBM tissue. NKCC1 colocalized with stem cell (Nestin), astrocyte (S100b), and neuronal progenitor cell (DCX) markers, but not with mature neurons (NeuN), consistent with its role in developmental and progenitor cell populations [25].

Overall, these results indicate a multifaceted role of NKCC1 in GBM biology, particularly in tumor progression and recurrence. They support further investigation into targeting chloride homeostasis as a therapeutic strategy in GBM and provide a rationale for developing selective *NKCC1* inhibitors and evaluating the effects of modulating the NKCC1/KCC2 balance *in vivo*.

The interpretation of OS in this study is constrained by the limited availability of clinical annotation. Survival information was accessible only for a subset of patients from whom tumor tissue was collected, and comprehensive data on key covariates, such as treatment regimens and extent of surgical resection, were not consistently available. As a result, multivariable analyses adjusting for potential confounding factors could not be performed. Furthermore, the OS analysis is likely underpowered due to the relatively small number of patients, and the absence of a statistically significant association between NKCC1 expression and OS should therefore be interpreted with caution. These limitations underscore the need for future studies in larger, well‐characterized cohorts to more definitively evaluate the prognostic relevance of NKCC1 in GBM.

## 5 Conclusions

This study offers new insights into the molecular heterogeneity of GBMs and identifies NKCC1 as a key regulator of tumor progression. The observed age‐related increase of *NKCC1* underscores the importance of personalized treatment strategies that consider patient‐specific molecular profiles. These findings suggest several promising avenues for future research, including the development of selective NKCC1 inhibitors with enhanced clinical applicability. In summary, this work advances our understanding of GBM biology and provides a foundation for developing more effective therapeutic approaches against this highly aggressive brain tumor.

## Conflict of interest

The authors declare no conflict of interest.

## Author contributions

A.T., D.F., M.H., S.K., C.S., and F.S. contributed to the data acquisition, data curation, and writing. C.S. and F.S. contributed to the review and editing, and final approval. S.K and D.F. contributed to the conceptualization, writing, review, and editing. All authors approved the final submitted version of the manuscript.

## Supporting information


**Fig. S1.** Impact of age on recurrence‐free survival.
**Table S1.** Patient characteristics and survival with molecular and clinical parameters (MGMT, IDH, sex, and location).

## Data Availability

Data are available within the article and/or the supplementary information.
