# Supplementary material for: NKCC1: A key regulator of glioblastoma progression
Source: Mol Oncol. 2026 Mar 24;20(6):1420–8. doi: 10.1002/1878-0261.70242 (PMC13238877; doi:10.1002/1878-0261.70242)
Supplement: Supplementary file 1 — Fig. S1. Impact of age on recurrence‐free survival. Table S1. Patient characteristics and survival with molecular and clinical parameters (MGMT, IDH, sex, and location). [file MOL2-20-1420-s001.docx]

**Supplementary material**

**Recurrence (month)**

0

2

4

6

8

10

12

14

16

**Survival properties**

0

0.2

0.4

0.6

0.8

1.0

Younger patients

Older patients

Older censored

Younger censored

**Fig. S1. Impact of age on recurrence-free survival**

Kaplan–Meier analysis showed that age had no statistically significant influence on recurrence-free survival at 9 months (p = 0.109). Recurrence-free survival did not depend on age, in contrast to overall survival, where younger patients survived significantly longer than older patients. To verify whether the statistically significant effect of NKCC1 on recurrence-free survival was independent of age, we performed a Kaplan–Meier analysis stratified by age. Patients aged ≤60 years (n = 16) were compared with patients >60 years (n = 32). This analysis revealed no significant difference between the two age groups (log-rank test, p = 0.109), indicating that the influence of NKCC1 on recurrence-free survival is independent of patient age.

**Table S1** Patient characteristics and survival data in relation to molecular and clinical parameters (MGMT, IDH status, sex, tumor location) and the study analyses in which the samples were used (1: NKCC1 in relation to clinical parameters, 2: KCC2 in relation to clinical parameters, 3: recurrence-free survival)

| **Serial number** | **Age [years]** | **Overall survival [months]** | **Recurrence-free survival [months]** | **Sex** | **Location** | **MGMT-methylation** | **IDH-status** | **Experiment** |
| --- | --- | --- | --- | --- | --- | --- | --- | --- |
| 1 | 44 | 32 | 11 | male | occipital right and frontal left | not methylated | wild type | 1,3 |
| 2 | 45 | 11 | 8 | male | occipital right and frontal left | not methylated | wild type | - |
| 3 | 45 | 8 | 5 | male | left frontal | not methylated | wild type | 1,2,3 |
| 4 | 48 | 9 | unknown | male | right okzipital | not methylated | wild type | 1,2 |
| 5 | 52 | 65 | 3 | male | temporal | methylated | wild type | 1,2,3 |
| 6 | 52 | 21 | 14 | female | temporal | methylated | wild type | 1,2,3 |
| 7 | 52 | 21 | 12 | female | right parieto-occipital | methylated | wild type | 1,2,3 |
| 8 | 53 | 18 | 7 | male | left temporal | not methylated | wild type | 1,2,3 |
| 9 | 53 | 6 | 0 | male | right temporal | not methylated | wild type | 1,2,3 |
| 10 | 53 | 19 | 10 | male | links temporal | not methylated | wild type | 1,3 |
| 11 | 54 | 14 | 8 | female | temporal | methylated | wild type | 1,2,3 |
| 12 | 54 | 25 | 8 | female | right temporal | methylated | wild type | 2 |
| 13 | 56 | 0.25 | 0 | male | left frontal medulla, precentral | methylated | wild type | - |
| 14 | 56 | 3 | 1 | male | right frontobasal | methylated | wild type | 1,2,3 |
| 15 | 58 | 27 | 4 | female | right frontal | methylated | wild type | 1,2,3 |
| 16 | 59 | 9 | 5 | male | right parieto-occipital | not methylated | wild type | - |
| 17 | 59 | 16 | 10 | male | left temporal | not methylated | wild type | 1,2,3 |
| 18 | 59 | 8 | 8 | male | right frontotemporoparietal | not methylated | wild type | 1,2,3 |
| 19 | 59 | - | 3 | male | right frontoparietal multifokal | not methylated | wild type | 1,2,3 |
| 20 | 59 | 8 | 5 | female | left frontal | methylated | wild type | 1,2,3 |
| 21 | 59 | - | 0 | female | Temporo-parieto-occipitale right | not methylated | wild type | 1,2,3 |
| 22 | 60 | 13 | 13 | male | left temporoparietal | not methylated | wild type | 1,2,3 |
| 23 | 61 | 9 | 0 | male | right temporal | not methylated | wild type | - |
| 24 | 61 | 9 | 4 | male | left parietal | methylated | wild type | 1,2,3 |
| 25 | 61 | 5 | 0 | male | right okzipital | not methylated | wild type | 1,2,3 |
| 26 | 62 | - | 1 | male | left parietofrontotemporal | methylated | wild type | 1,2,3 |
| 27 | 63 | 29 | 11 | female | left temporo-occipital | not methylated | wild type | 2 |
| 28 | 63 | - | 4 | male | left temporal | methylated | wild type | 1,2,3 |
| 29 | 64 | 6 | 11 | female | right temporal and parietal | not methylated | wild type | 1,2,3 |
| 30 | 64 | 3 | 5 | female | left parietookzipital | not methylated | wild type | 1,2,3 |
| 31 | 64 | 72 | 6 | female | right parieto-occipital | methylated | wild type | 1,3 |
| 32 | 65 | 3 | 0 | female | right temporo-parietal | methylated | wild type | 1,2,3 |
| 33 | 65 | 3 | unknown | female | right temporal | methylated | wild type | 1,2 |
| 34 | 66 | 11 | 7 | male | left frontoparietal | not methylated | wild type | 2 |
| 35 | 66 | 6 | 3 | female | frontal left and right | not methylated | wild type | 1,2,3 |
| 36 | 66 | 12 | 7 | male | right frontal | not methylated | wild type | 1,2,3 |
| 37 | 69 | 10 | 4 | female | right parieto-temporo-okzipital | methylated | wild type | 1,2,3 |
| 38 | 71 | 8 | 0 | female | left frontal | methylated | wild type | 1,2,3 |
| 39 | 71 | 4 | 0 | female | left frontotemporal  and right temporal | methylated | wild type | 1,2,3 |
| 40 | 71 | 3 | 0 | male | right temporal | methylated | wild type | 1,2,3 |
| 41 | 71 | 14 | 4 | female | right temporal | not methylated | wild type | 1,2,3 |
| 42 | 71 | - | 2 | female | left frontal | not methylated | wild type | 1,2,3 |
| 43 | 72 | - | 3 | male | right frontal | not methylated | wild type | 1,2,3 |
| 44 | 73 | - | 12 | male | parietal | methylated | wild type | 2 |
| 45 | 73 | - | 5 | male | right frontal | methylated | wild type | 1,2,3 |
| 46 | 73 | - | 0 | female | right frontoparietal | methylated | wild type | 1,2,3 |
| 47 | 73 | 17 | 9 | female | right temporal | not methylated | wild type | 1,2,3 |
| 48 | 74 | - | 5 | male | right frontal | methylated | wild type | 1,2,3 |
| 49 | 74 | - | 7 | female | right frontal | methylated | wild type | 1,2,3 |
| 50 | 75 | - | 8 | male | right parietal | methylated | wild type | 1,2,3 |
| 51 | 77 | - | 0 | female | left frontal | methylated | wild type | 1,2,3 |
| 52 | 77 | 2 | 0 | female | left frontal | methylated | wild type | 1,2,3 |
| 53 | 77 | 3 | 0 | female | temporal | not methylated | wild type | 2 |
| 54 | 78 | - | 5 | male | left temporal | methylated | wild type | 1,2,3 |
| 55 | 79 | 3 | 0 | female | right frontoparietal | not methylated | wild type | 1,2,3 |
| 56 | 79 | 11 | 2 | male | left frontal | methylated | wild type | 1,2,3 |
| 57 | 80 | 6 | 2 | female | right frontal | methylated | wild type | 1,2,3 |
| 58 | 80 | 0,25 | 0 | male | right frontotemporoparietal | methylated | wild type | - |
| 59 | 81 | 4 | 0 | male | parieto-occipital left | methylated | wild type | 1,3 |
| 60 | 82 | - | 0 | female | left parietal | not methylated | wild type | - |
| 61 | 82 | - | 0 | male | left parieto-occipital | methylated | wild type | 1,2,3 |
